# Model for building quality resilient health facility

**DOI:** 10.3389/fpubh.2023.1269330

**Published:** 2023-12-01

**Authors:** Elom Hillary Otchi, Nhyira Gyawu, Gilbert Buckle

**Affiliations:** ^1^Africa Institute of Healthcare Quality Safety and Accreditation (AfIHQSA), Accra, Ghana; ^2^Korle Bu Teaching Hospital, Accra, Ghana

**Keywords:** quality, health system, quality improvement, accreditation, standards

## Abstract

The AfIHQSA Model is the model for building quality resilient health systems. It is proposed as a compliment to and in many instances as an alternative to the many other existing in ensuring a systematic and a sustained approach to improving outcomes in African health systems. It seeks to bring the necessary transformation to healthcare quality and patient safety and facilitate the attainment of desired outcomes. The model is unique in its iterative nature and how it places premium on sustaining the gains of improvement. The authors are concerned about the lack of sustainability of the many quality improvement efforts on the continent and how they all fade out into obscurity upon the exit of the proponents. Six iterative steps are proposed in the use of the model and these are: leadership commitment and buy-in; situational analysis of quality management capacity; systems strengthening for quality management; quality improvement interventions for care outcomes; standardization/accreditation/certification; and iterative monitoring, evaluation of performance of interventions and learning. Most of the quality interventions and efforts on the continent have failed because the steps in this model have not been sufficiently followed and addressed. The required strengthening of the various components of the health system necessary to sufficiently bear the weight of any quality intervention and guarantee sustainability of the gains is often ignored. As authors, we have therefore formally adopted the use of this model and plan to further continue evaluating and monitoring its utility and its generalizability in different institutions and countries.

## Background

As healthcare and its practice continues to evolve, the concept of quality remains extremely relevant and an incessant topic of interest today. It has been identified as the critical challenge confronting health systems especially in Africa (1) and perhaps the single most important requirement for the attainment of Universal Health Coverage (UHC). The WHO Director-General was apt in indicating for instance that, *“without quality, the UHC remains an empty promise…unless those services are of sufficient quality”* (2).

Quality has been defined as “the extent to which health services for individuals and the populations increase the likelihood of desired care outcomes and is consistent with current professional knowledge” (3). Eight (8) dimensions (i.e.*, safety, timeliness, efficient, effective, equitable, person*-centered *care, integrity and integrated*) of healthcare defines its quality (4–6). Unfortunately, the quality of care across the world and particularly in Africa do not meet these dimensions (7, 8). In view of the above, there have been various propositions and “how-to’s” to improve global health systems especially in LMICs and Africa. The WHO recently launched a planning guide for quality health services which among others highlight the need for a health system approach in implementing key activities for improved quality of care outcomes (9). There have also been a proliferation of models and frameworks on measuring and improving the quality of healthcare (10–13). However, the global and more importantly, the quality situation in Africa does not seem to be improving as expected inspite of the various efforts including the development and implementation of national quality strategies across the continent and support by donors and other international agencies/partners.

In addition, and unfortunately, there also seem to be no consensus on the appropriate quality model/framework to guide African health systems toward improvement. Varied quality models and frameworks have been proposed (9, 12–14) as the panacea to attaining improved outcomes of care especially in African health systems. Inspite of the proliferation and use of these models, there is very little evidence of their effectiveness in improving outcomes of care especially in Africa (15). Many countries on the continent continue to pilot varied improvement ideas several years after the implementation of various quality programs and models/frameworks. The desired improvement is still yet to be realized. In countries where there were any glimpses of hope, these improvements have not been sustainable, lending credence to the fact that most of the existing quality frameworks/models have proven insufficient in addressing the complexities in African health systems. The continuous use of these quality models in their present states will make it impossible for African health systems to either attain the desired improvements or meet the targets of the SDGs. The authors define a quality resilient health system/facility as one that is able to withstand and/or immediately recover from health systems’ shocks and emergencies as was experienced during the COVID-19 pandemic. Some countries in Africa are still yet to recover from the devastating effects of the pandemic.

## Design of the model

The model was designed informed by literature of the most common frameworks/models used for quality improvement in African health systems; and the interaction of the authors with various health systems on the continent. The process was iterative and involved the documentation of the experiences of the authors in their efforts to improve the quality of care outcomes across the many health facilities on the continent. Several models and techniques for performing QI have been developed although many share underlying principles, including, identifying the quality issue, understanding the problem from a range of perspectives, with a particular emphasis on using and interpreting data, developing a theory of change, identifying and testing potential solutions, using data to measure the impact of each test and gradually refining the solution to the problem, implementing the solution and ensuring that the intervention is sustained as part of standard practice [38]. The AfIHQSA model offers a structured and a more sustainable approach to improving care outcomes in Africa. The model provides consistency and a common thinking across healthcare organizations/systems. Most of the existing models in use in Africa such as Six Sigma, Lean, Model for Improvement, PDSA etc. have all proven insufficient in addressing the complexities of African health systems, created a lot of confusion and a barrier to the uptake of quality improvement (3).

## The AfIHQSA model

The model for building quality resilient health facility addresses the current gaps such as lack of leadership involvement, accountability and support; lack of sustainability of quality interventions with credible and sound evidence base; inadequate understanding of the context by improvers; weak health systems that are incapable of carrying the weight of and sustaining improvement; and varied improvement models/approaches that only end up confusing healthcare providers and organizations (16–19) and the inability to appreciate and see quality as part of the complex adaptive system of healthcare (20) among others. It is grounded on the fact that, in planting the seed for quality, the ground has to be fertile and ready. It is mindful of the fact that, a health system has to be resilient toward sustaining the implementation of quality practices and achievement of quality outcomes. We define resilience as a health system that is sensitive and responsive to deviations from the desired quality outcomes. A quality-sensitive health system is thus one that is innately able to identify the quality needs of the population (i.e.*, users and providers in the facility or country*) while a quality-responsive health system is one that is able to resource and continuously execute quality improvement practices carried out in the health facility. These two attributes of sensitivity and responsiveness are mutually reinforcing. An increasingly quality-sensitive health system becomes increasingly quality-responsive and the reverse is true as well.

**Figure 1:**
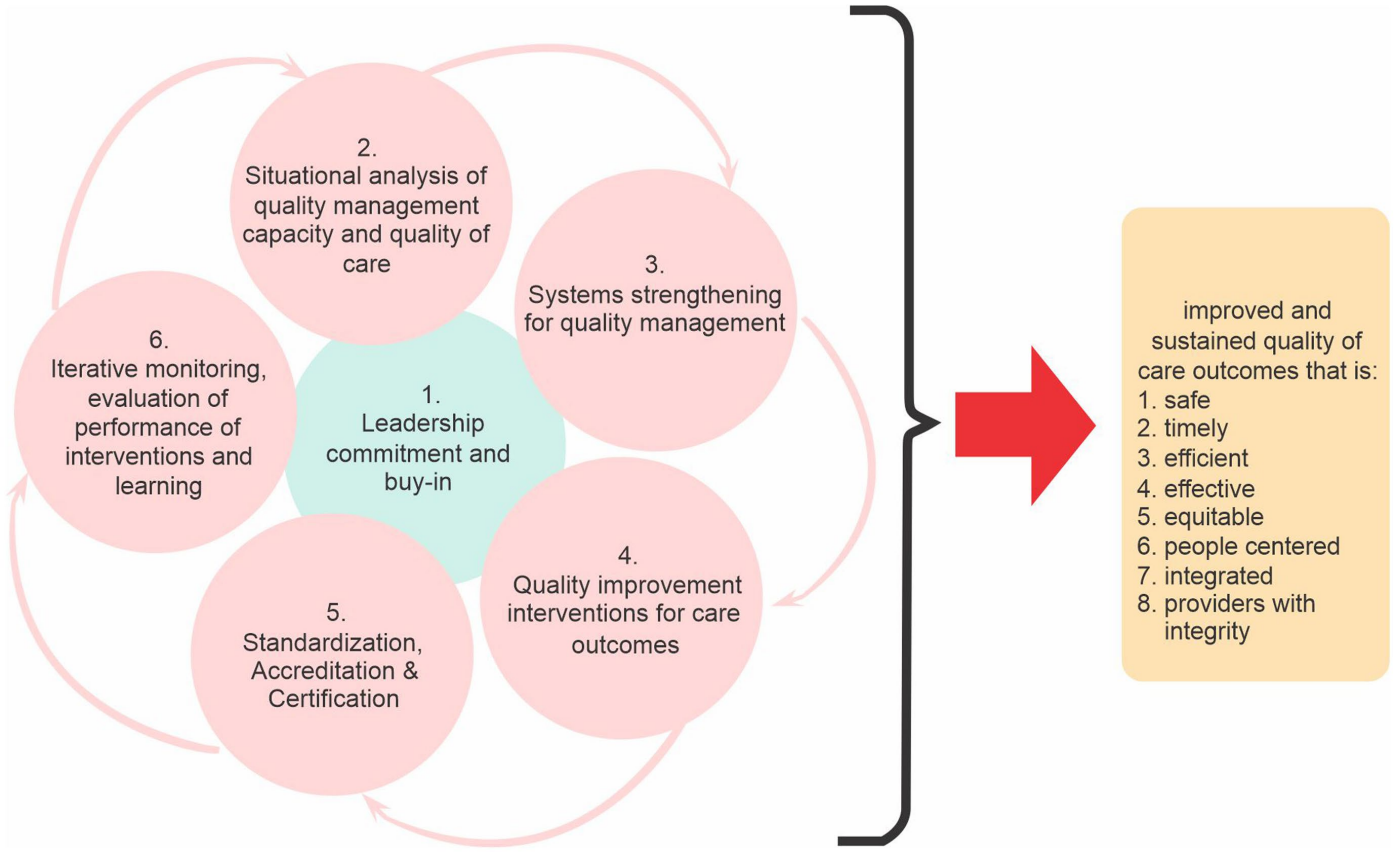
AfIHQSA model for building quality resilient health facility.

The model guides in the determination and selection of the appropriate quality metrics, method/approach and interventions, and facilitates the documentation of improvement efforts in the health facility. It has six (6) cyclical and interrelated stages (Figure 1). The first stage is securing and ensuring leadership (top management) commitment, ownership and buy-in. This is one of the most important and critical success factors for achieving a high-quality performing organization. Various authors have identified the lack of support and commitment from top management/leadership as one of the main reasons why improvement efforts fail (21, 22). This stage is an iterative and a never-ending process which permeates all the other 5-phases of the model. The authors are of the opinion that, without leadership (top management) appreciation, understanding and commitment to an organizational culture toward quality, very little and in some instances, nothing will be achieved. As much as possible, top management and key actors in the process must see this as “ours” other than “theirs” in every stage of the cycle. The leadership that is required for a successful implementation of this model is one that is humble, accountable, collaborative, willing to learn, fully aware of the evolving complexities of the health system and relationships (*of providers, patients etc.*), honest, full of integrity, emotionally intelligent, systems thinker and one that is willing and able to work with all stakeholders to ensure and sustain improvement.

The context in which quality is to be achieved is very important and unique to each organization (22). Therefore, knowledge of the current situation in the organization or country is fundamental to initiating any form of intervention. In looking at the current situation, both the capacity of the health system to deliver quality outcomes and the level of quality needs to be assessed. There is the need to establish the extent of quality readiness in the organization or country. A situational analysis of the country healthcare organization’s quality management capacity and quality of care is therefore essential. Further, the situational analysis should also assess the strength and resilience of the various components of the health system and make a determination of the enablers and barriers to quality. Is the weight of the current health system well able to carry and sustain any quality improvement intervention(s)? Is the health system ready to carry on any additional responsibility? These help to determine where to pluck in and effectively intervene. This is an important factor in the process to determine how to proceed and what modifications ought to be made to any intended interventions. Unfortunately, often times, improvers are in a hurry to intervene and they ignore all the necessary first-steps that will enable them to have a deep understanding and appreciation of the context and the terrain. This is a very patient and meticulous step in the stages of this model because it is invariably one of the key determinants of the level of sustainability or otherwise of any quality intervention.

Strengthening the respective components of the health system for quality management is the third stage in the model. This component of the model is consistent with the WHO’s Health Systems Framework (23). The foundation of quality is a health system designed for quality, as such the model proposes the need to strengthen the facility’s health systems components to better manage and implement quality. There is the need to ensure that, all the nine (9) health systems building blocks (*leadership/governance, human resource, service delivery, health finance, health technology, health information, community participation, partnership and research*) (24) are in synch and sufficiently strengthened for quality management. This is necessary because, “working in health systems that are inefficient and irritating has a negative [effect] on one’s ability” (25, 26) to deliver quality care outcomes. You need a robust hospital management information system (HMIS) to measure improvement, you need well-motivated and capacitated human resource at the front line. The required funding also has to be made available to facilitate the purchase of the necessary resources to make improvement possible. Health systems leaders in Africa should work toward increasing their health expenditure to the recommended 15% (27). In some health systems, availability of competent human resource is a big challenge (28, 29), hence improvement efforts revolve around a few members of staff whose absence jeopardizes the entire quality program. We are of the opinion as authors that, effective implementation and sustainability of any improvement effort depends on a strengthened health system. Improvers should therefore expend a lot of energies in strengthening the building blocks prior to the introduction of any improvement intervention. Until this is sufficiently targeted and addressed, sustainability of any improvement intervention is not guaranteed. We have so many countries and institutions implement quality interventions on the back of very weak health systems (18, 30), in such instances, the interventions become unsustainable.

When the system is ‘quality ready’, quality improvement interventions are then initiated, “tested” and subsequently implemented to achieve improved service and care outcomes. This is the fourth stage of the model. All the quality models identified and discussed in previous sections of this paper can be plucked in here with the assumption that the improver has sufficiently gone through and addressed all the issues in the previous stages, and have sufficiently identified strategies to address their weaknesses as well. Improvers should be mindful of the weaknesses of the various quality models in their application. Unfortunately, and in many instances, improvers rather pluck in here because their sponsors are in hurry to see results. Therefore, without much understanding of the context, so many improvement projects are initiated only to fail after the project life cycle or the exit of the sponsor(s). In one of the countries that one of the authors supported, healthcare workers were being paid monthly stipends for undertaking their quality improvement (QI) projects. Unfortunately, the Ministry of Health (MoH) could not continue/sustain this approach after the sponsors exited. The question that we ask is, why should improvers and sponsors use money to entice healthcare workers to do quality improvement when they well know that this is not sustainable? Why do they cause such disruptions in Africa & LMICs health systems when they well know ministries of health will not be in a position to continue funding such an expenditure outside what is mandated and approved by the national governments? And why should Africa or LMIC health systems allow what will ordinarily not be allowed in other health systems? Improvers should always take the admonishing of Paul Bataldan who said, “everyone in health care has two jobs: to work and to improve it.” The authors propose therefore that, improvement interventions should be one that will ensure improved outcomes in the short to long-term, will not negatively affect any component of the health system and will be sustainable even after the exit of the funders.

Once sufficient evidence about the effectiveness of the interventions have been adduced, we propose that they should be scaled up and standardized across the health system to eliminate variation. Having worked in various health systems such as Ghana, Liberia, Malawi, Sierra Leonne and Botswana; we are yet to see many sustained improvement projects whose processes have been standardized and scaled up. Here, we propose that, the evidence and learning should be scaled up and the processes should be standardized. We define standardization as the method of establishing a specific policies and practices that are recognized and acts as a guideline or a model for a process (13). Standards or best practices are therefore the actual documented policies, methods, equipment, and training (13). Where healthcare organizations can, independent parties should be invited in the form of certification or accreditation programs to further assess or evaluate and endorse their efforts.

The final stage of the model is the iterative monitoring, evaluation and learning to feedback and continuously improve the intervention design and execution. Unfortunately, we are of the opinion as authors that, many have been preoccupied with the stage of improvement interventions to the neglect of the various sub-components of the model which are very fundamental in ensuring sustainability. The authors are further of the opinion that, this model is a sure way of ensuring sustainability of any quality improvement effort in any health facility in Africa because it is very cognizant of the role context and how it influences any change effort.

We have observed from our practice in 5 countries and working in more than 200 health facilities of all sizes (tertiary to clinics) that often times, health facilities start quality improvement interventions at points when the health system building blocks are less resilient to begin any “serious” quality journey. In such instances, the system soaks the pressure for a while especially when the sponsors are still around and giving all the necessary support. However, the system inevitably gives up because of the inherent weaknesses and barriers; and these become markedly evident when the sponsors have left. It is suggested for instance that, many PDSA published projects had limitations with their design, incomplete reporting and inadequate data analysis (11, 15, 22). Improvement efforts could also be extremely chaotic, less effective and unlikely to achieve its intended outcomes without a model that is systematic in its approach and mindful of the nuances within the health facility/system where the change is intended.

The authors believe and are of the opinion that, the model, if applied, is iterative and responsive in determining the readiness and possible success of a health facility’s quality readiness status. It is an important advancement over other models that are only “quality improvement interventions” oriented without an appropriate consideration to key and relevant health systems factors that will ensure sustainable improvement efforts. In applying the model, one has to be mindful of the need to develop team cohesion, individual capacity to innovate and initiate change, health workers’ intrinsic motivation to achieve high levels of performance and commitment to quality. The involvement of key stakeholders in the planning and execution of activities is a critical success factor. One of key assumptions to the use of this model is that, African or LMICs health systems are receptive to change.

## Conclusion and recommendations

We conclude that, the model is a better fit of the reality of the African context and situation. It reflects the reality of Africa health systems and offers a solution that is consistent to the desires and aspiration of the many patients that uses healthcare on the continent. The model, if used as described, will ensure that, quality of care outcomes is sustainable. One of the main strengths of this model is that, it can be used together with other models. Further, leadership is key in ensuring a successful outcome. The leadership that is not required is one that pays lip service, absent, disinterested, uncommitted and lacks integrity. A health system that is resilient is a requisite for quality improvement interventions and sustained quality efforts. An understanding of the health system is required to define the direction of any change or improvement initiative.

The improver that is required for a successful use of this model is one that is emotionally intelligent, versatile, a systems thinker, bold, humble, has a sense of integrity and is skillful in facilitation and effective communication will be able to easily reap the benefits of the application of this model. It has performed well in sustaining the improvement efforts in the various healthcare facilities where it was applied by the authors. We as authors have therefore formally adopted this model as the *“AfIHQSA Model for Building Quality Resilient Health Facility”* in supporting healthcare facilities to improve their outcomes of care. We plan to further continue evaluating and monitoring its utility and its generalizability in different institutions and countries.

## Strengths and limitations of the AfIHQSA

The following are the strengths of the AfIHQSA Model over other models. These strengths address the gaps in other quality models:

1. It can be used together or alongside other quality improvement models
2. It compliments corporate or organizational operations and ensures that the total organizational change results in cost and time savings unlike other quality models
3. This model is team-dependent other than individual-dependent, hence the absence of a team member does not in any way impact negatively on its utility.
4. The AfIHQSA Model is less expensive to implement
5. The AfIHQSA Model enhances and/or encourages creativity and ensures that this permeates the culture of the organization
6. Further, the Model is adaptable and can be used in any kind of health system
7. Finally, the AfIHQSA Model recognizes, considers and acknowledges the inherent challenges of every health system such as its complexity, sophisticated technology, marked inter- and intra-professional turf war, and inadequate infrastructure and data use for decision-making in its implementation unlike other quality models.

The limitations of this model include the following:

1. The various knowledge requirements in the application of the Model to ensure success.

## Data availability statement

The original contributions presented in the study are included in the article/supplementary material, further inquiries can be directed to the corresponding author.

## Author contributions

EO: Conceptualization, Methodology, Validation, Writing – original draft, Writing – review & editing. NG: Formal analysis, Methodology, Writing – original draft. GB: Conceptualization, writing – original draft, Writing – review & editing.
